# Gestational high-fat diet and bisphenol A exposure heightens mammary cancer risk

**DOI:** 10.1530/ERC-17-0006

**Published:** 2017-05-09

**Authors:** Yuet-Kin Leung, Vinothini Govindarajah, Ana Cheong, Jennifer Veevers, Dan Song, Robin Gear, Xuegong Zhu, Jun Ying, Ady Kendler, Mario Medvedovic, Scott Belcher, Shuk-Mei Ho

**Affiliations:** 1 Department of Environmental HealthCincinnati, Ohio, USA; 2 Center for Environmental GeneticsUniversity of Cincinnati College of Medicine, Cincinnati, Ohio, USA; 3 Cincinnati Cancer CenterCincinnati, Ohio, USA; 4 Department of Pathology and Laboratory MedicineUniversity of Cincinnati College of Medicine, Cincinnati, Ohio, USA; 5 Department of Pharmacology and Cell BiophysicsUniversity of Cincinnati College of Medicine, Cincinnati, Ohio, USA; 6 Cincinnati Veteran Affairs Hospital Medical CenterCincinnati, Ohio, USA

**Keywords:** breast cancer, developmental origin of health and disease (DOHaD), RNA-seq, transcriptomics, DNA methylation, patient survival, TCGA, windows of susceptibility, *in utero* exposure, bisphenol A, high-butter fat diet, non-monotonic response

## Abstract

*In utero* exposure to bisphenol A (BPA) increases mammary cancer susceptibility in offspring. High-fat diet is widely believed to be a risk factor of breast cancer. The objective of this study was to determine whether maternal exposure to BPA in addition to high-butterfat (HBF) intake during pregnancy further influences carcinogen-induced mammary cancer risk in offspring, and its dose–response curve. In this study, we found that gestational HBF intake in addition to a low-dose BPA (25 µg/kg BW/day) exposure increased mammary tumor incidence in a 50-day-of-age chemical carcinogen administration model and altered mammary gland morphology in offspring in a non-monotonic manner, while shortening tumor-free survival time compared with the HBF-alone group. *In utero* HBF and BPA exposure elicited differential effects at the gene level in PND21 mammary glands through DNA methylation, compared with HBF intake in the absence of BPA. Top HBF + BPA-dysregulated genes (*ALDH1B1*, *ASTL*, *CA7*, *CPLX4*, *KCNV2*, *MAGEE2* and *TUBA3E*) are associated with poor overall survival in The Cancer Genomic Atlas (TCGA) human breast cancer cohort (*n* = 1082). Furthermore, the prognostic power of the identified genes was further enhanced in the survival analysis of Caucasian patients with estrogen receptor-positive tumors. In conclusion, concurrent HBF dietary and a low-dose BPA exposure during pregnancy increases mammary tumor incidence in offspring, accompanied by alterations in mammary gland development and gene expression, and possibly through epigenetic reprogramming.

## Introduction

Breast cancer is a major global public health problem and effective prevention strategies are needed to combat the disease, most desirably at the primary prevention level (Hanf & Gonder 2005, Fabian *et al*. 2015). In addition to family history, other risk factors including hormonal and environmental factors, particularly nutrition, have been identified to be major contributors in the etiology of breast cancer (Peto 2001, Holmes & Willett 2004, Adebamowo *et al*. 2005, Teegarden *et al*. 2012). For example, exposure to Western lifestyles dramatically increases breast cancer incidence in Asian immigrants in the United States (US) during their lifetime and in their offspring over several generations (Buell 1973, Ziegler *et al*. 1993). Maternal exposures to endocrine-disrupting chemicals (EDCs) and certain dietary factors during pregnancy have been reported to be associated with increased mammary tumorigenesis among female offspring (Prentice *et al*. 2006, Durando *et al*. 2007, Moral *et al*. 2008, Doherty *et al*. 2010, Soto *et al*. 2013). However, cohort and human case–control studies have generated conflicting reports regarding the effect of high-fat intake on adult breast cancer risk (Hulka 1989, Willett *et al*. 1992, Hunter *et al*. 1996, Martin *et al*. 2011), partly due to differences in fat/dietary composition, and study design, including exposure window. For example, consumption of animal fat, consisting mainly of saturated fatty acids, is associated with increased risk of breast cancer in some studies (Cho *et al*. 2003, Gonzalez & Riboli 2010) but not in others (Lof *et al*. 2007, Key *et al*. 2011, Park *et al*. 2012). The development of rodent models have proved critical to assess the effects of single and/or multiple exposures on susceptible windows of mammary gland development and in the subsequent identification of factors relevant for breast cancer prevention in humans.

The mammary gland has been shown to be particularly sensitive during early development, both to dietary fatty acids and EDCs such as bisphenol A (BPA), perhaps because extensive programming of the mammary gland occurs during fetal life (Hilakivi-Clarke *et al*. 1995, 1996, 1999, 2002, Durando *et al*. 2007, Moral *et al*. 2008, Lo *et al*. 2009, Doherty *et al*. 2010, MacLennan & Ma 2010, Soto *et al*. 2013). High-fat intake during pregnancy has been shown to increase offspring’ mammary cancer risk by altering mammary gland development, mainly by increasing the number of terminal end buds (TEBs) (Hilakivi-Clarke *et al*. 1997, de Assis *et al*. 2006), the purported target structures of malignant transformation (Russo *et al*. 1983, Welsch 1985, Russo & Russo 1996*a*,*b*). Similarly in rodent models, BPA has been reported to alter the development of the mammary gland suggesting the possibility of increased susceptibility to mammary tumorigenesis (Markey *et al*. 2001, Munoz-de-Toro *et al*. 2005, Durando *et al*. 2007). Using the 7,12-dimethylbenz(a)anthracene (DMBA)-induced mammary carcinogenesis Sprague–Dawley (SD) rat model, Russo and coworkers (Betancourt *et al*. 2010) reported that a prenatal BPA dose of either 25 or 250 μg/kg body weight (BW)/day by itself had no tumorigenic effect after DMBA exposure on postnatal day (PND)50. However, when DMBA was administered at PND100 following prenatal BPA exposure at 250 µg/kg BW/day, the EDC exposure resulted in a significantly increased number of terminal ducts (Moral *et al*. 2008) as well as a higher incidence of mammary tumors (Betancourt *et al*. 2010). As the epigenome is most susceptible to perturbations in early development, the adverse effects of *in utero* exposures on adult health are likely mediated by epigenetic dysregulation of gene expression (Perera & Herbstman 2011). Indeed, a high-fat- or ethinylestradiol-supplemented maternal diet has been shown to increase mammary cancer risk in several generations of offspring and is associated with changes in the DNA methylation machinery and methylation patterns in mammary tissue (de Assis *et al*. 2012).

We are not aware of any previous studies that have investigated the impact of maternal consumption of a high-butterfat (HBF) diet together with exposure to low-dose BPA on mammary cancer risk of offspring. The present study aimed to test the hypothesis that maternal exposure to low, environmentally relevant doses (2.5–2500 µg/kg BW/day) of BPA, in addition to HBF intake during pregnancy leads to increased incidence of mammary cancer in offspring. According to our previous study, we chose butter as the source of high fat (39% kcal) to mimic a key fat component in the Western diet, and which does not promote obesity in our animal model (Medvedovic *et al*. 2009). Our results indicate that concurrent exposure of dams to a HBF diet and BPA at 25 μg/kg BW/day dose level during pregnancy increases mammary tumor incidence in offspring treated with DMBA on PND50, accompanied by alterations in mammary gland development. Furthermore, *in utero* HBF and BPA exposure elicited differential effects at the gene level in prepubertal mammary glands through DNA methylation, compared with HBF in the absence of BPA. A signature of top dysregulated genes was subsequently found to be associated with poor overall survival in populations of breast cancer patients from The Cancer Genomic Atlas (TCGA). These findings highlight the importance of future studies to address maternal diets as modifiers of susceptibility to *in utero* exposure to environmental agents for devising new strategies to reduce breast cancer risk.

## Materials and methods

### Animals

Female, virgin SD rats at ~7 weeks of age were obtained from Taconic Farms (Germantown, NY, USA). Animals were housed individually in a temperature- and humidity-controlled environment with a 12-h light–darkness cycle, in the AAALAC-approved University of Cincinnati animal facility. All rats were provided food and filtered water *ad libitum*, and animals were housed on sani-chips bedding and maintained in an environment under controlled endocrine-disrupting chemical (EDC) exposures (Thigpen *et al*. 2013, Martinez *et al*. 2015). All animal procedures were approved by the University of Cincinnati Institutional Animal Care and Use Committee, and experiments were performed following the guidelines of the National Institutes of Health for the proper and humane use of animals in biomedical research.

### Experimental design and mammary tumorigenesis

**Figure 1:**
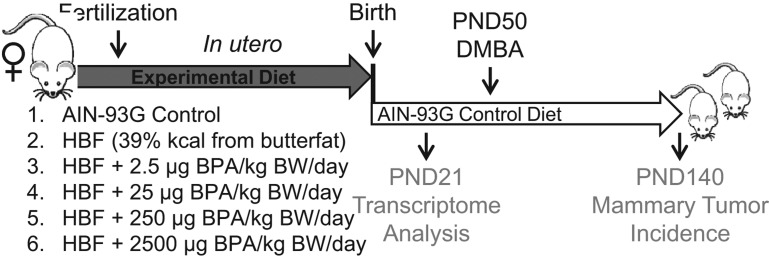
Schematic diagram of the experimental design. Female SD rats (7–9 weeks old) were randomized into 6 groups (*n* = 11 litter/group, one offspring/litter). During mating and throughout gestation, dams were fed a control AIN-93G diet or a modified AIN-93G high-butterfat (HBF) diet in the presence or absence of bisphenol A (BPA) at various concentrations (µg/kg BW (body weight)/day). After birth, dams and offspring were maintained on an AIN-93G diet for the duration of the experiment. Pups were weaned at PND21 and one female offspring per litter was killed for mammary gland transcriptome analysis. At PND50, one female offspring from each dietary group was treated with a single oral dose (20 mg/kg BW) of DMBA to induce mammary cancer. All pups were killed at PND140 for analysis and determination of mammary tumor incidence.

Female SD rats (7–9 weeks old) were randomized into 6 groups (*n* = 11). After two weeks (acclimation) of experimental diet group exposure, female rats were bred with male SD rats (~3 months of age). During mating and throughout gestation, dams were fed either a control AIN-93G diet (10% kcal from butterfat) or a modified AIN-93G high-butterfat diet (39% kcal from butterfat), in the presence or absence of BPA (Sigma-Aldrich) at various environmentally relevant doses: 2.5, 25, 250 or 2500 µg/kg BW/day. Diets were controlled for caloric content, vitamins, salts and protein, but varied in fat and carbohydrate content. After birth, dams and offspring were maintained on control AIN-93G diet for the duration of the experiment. Litters were weighed weekly until killed. The date of vaginal opening was recorded as an indication of reaching sexual maturity. At PND50, one female offspring per litter per group was treated with a single oral dose (20 mg/kg BW) of DMBA (Thermo Fisher Scientific) to induce mammary cancer, and another littermate was given corn oil (Sigma-Aldrich) as a control. Animals were palpated weekly to monitor tumor development. Tumor size was measured using a caliper and tumor volume was calculated. All pups were killed at PND140, or when tumor burden exceeded 10% of total BW. Time to first tumor appearance (latency), the number of animals with palpable tumors (incidence) and the number of tumors per animal (multiplicity) were determined. The origin of the tumor was confirmed by a clinical pathologist (AK). Serum hormone levels of 17β-estradiol (Calbiotech, Spring Valley, CA, USA), progesterone (IBL, Minneapolis, MN, USA), leptin (EMD Millipore) and adiponectin (EMD Millipore) at PND21 and 50 were measured using ELISA-based assays. A schematic diagram summarizing the experimental design of this study is presented in Fig. 1.

### Whole mount and morphometric analysis

The fourth abdominal mammary gland from PND21 female pups was prepared as whole mount for morphological analysis according to an established protocol (Russo & Russo 1978). The total number of terminal end buds (TEBs) was evaluated by counting individual structures under a dissection microscope.

### Laser capture microdissection

PND21 mammary glands from female offspring (*n* = 5 L/group, one offspring/L) fed a HBF diet and a HBF diet with 25 µg BPA/kg BW/day were chosen for laser capture microdissection (LCM) according to our published protocol (Zhu *et al*. 2004). Briefly, mammary glands were first cryosectioned with 10 micron thick, hematoxylin-stained and dried for microdissection. Multiple sections (~5) per mammary gland were microdissected using an Arcturus Veritas Laser Capture Microdissection System (Thermo Scientific).

### RNA sequencing

Total RNA was extracted from LCM samples using a Qiagen RNeasy Lipid kit (Qiagen). The RNA quality and quantity were assessed using Agilent Bioanalyzer (Agilent) and NanoDrop ND-1000 spectrophotometer (Thermo Scientific), respectively. RNA libraries were prepared according to manufacturer’s protocol of TruSeq RNA sample preparation kit (Illumina, San Diego, CA, USA) and were sequenced with Genome Analyzer II sequencing system in the Genomics, Epigenomics and Sequencing Core at the University of Cincinnati. Analysis of gene expression was performed with our standard pipeline (Govindarajah *et al*. 2016). Differentially expressed genes of the HBF + BPA group were selected based on *P* < 0.05 and a fold-change greater than 2, when compared with the HBF-alone group. Functional enrichment analysis of differentially expressed genes was performed using the knowledge-based Ingenuity Pathways Analysis (IPA) (Qiagen, www.qiagen.com/ingenuity). RNA-seq data were deposited in the NCBI Gene Expression Omnibus database with accession number GSE73604.

### Real-time PCR (qPCR) analysis

Total RNA extracted from microdissected samples was amplified using the RiboAmp HS PLUS RNA Amplification kit (Applied Bisosytems) according to the manufacturer’s protocol. RNA expression was semi-quantified by SYBR GreenER (Thermo Fisher Scientific) using the 7900HT Fast Real-time PCR System (Thermo Fisher Scientific). Primer sequences are listed in Supplementary Table 1 (see section on supplementary data given at the end of this article). The target gene expression was normalized with endogenous *Rpl19* level, and the relative change in transcript level was calculated using the delta-delta-CT method (Livak & Schmittgen 2001).

### Bisulfite sequencing analysis

Sections from PND21 mammary glands in female offspring (*n* = 4–6 litter/group, one offspring/litter) were used for genomic DNA (gDNA) extraction and for bisulfite sequencing analysis according to our published protocol (Zhu *et al*. 2004).

### TCGA survival analysis

RNA-seq analysis (RNA-seqV2) of The Cancer Genomic Atlas (TCGA) breast cancer samples as well as patient clinical data were downloaded (http://cancergenome.nih.gov) on 3/8/2016. The original TCGA breast cancer data set consists of RNA-seq data from 1215 samples. Data on tumor adjacent normal (113), metastatic tumor (7) and male breast cancer samples (12) were excluded. One sample was discarded due to lack of survival data. In this study, only 1082 female samples (746 Caucasian, 180 African American, 61 Asian, 1 American Indian or Alaska Native and 94 unknown) with ER status (795 ER+, 237 ER− and 50 indeterminate or N/A) were used for survival analyses. Normalized data of seven genes identified and verified in the LCM study (*ALDH1B1*, *ASTL*, *CA7* (official gene symbol for carbonic anhydrase VII in human), *CPLX4*, *KCNV2*, *MAGEE2* and *TUBA3E* (equivalent to *Tuba3A* in rat)) were variance stabilizing-transformed before hierarchical clustering with complete linkage based on the Euclidean distance between genes was performed to dichotomize the cohort into two groups. Survival data including days-to-last follow-up and days-to-death were extracted from TCGA clinical data. Overall survival between two groups was analyzed by Kaplan–Meier plot with log-rank test, and Cox proportional hazard models (with age at initial pathologic diagnosis adjusted) were used to analyze time to death. All analyses were carried out using survival package in R (http://www.r-project.org). To further determine the characteristics between the two groups, clinical features including age, estrogen receptor status, progesterone receptor status, cancer stage, cancer recurrence as well as lymph node positivity were compared using Student *t*-test (continuous variables) or Pearson’s chi square test with contingency tables (categorical variables). The tests were conducted using GraphPad Prism 5.0.

### Statistical analysis

Gene expression and bisulfite sequencing analyses were performed using GraphPad Prism 5.0. For gene expression analyses, data were expressed as mean ± standard error of mean (s.e.m.) and analyzed by Student *t*-test and *post hoc* Mann–Whitney test. Bisulfite sequencing data were expressed as % methylation and analyzed using two-way ANOVA and Bonferroni post-test. *P* < 0.05 was considered as statistically significant when compared between and among groups.

## Results

### Gestational high-fat intake in addition to bisphenol A exposure increases mammary tumor incidence and effects mammary gland morphology

To test our hypothesis that maternal exposure to BPA in addition to HBF intake during pregnancy leads to increased incidence of mammary cancer in offspring, we fed female SD rats (F0) with a modified AIN-93G HBF diet containing 39% kcal from butterfat, during mating and throughout gestation, in the presence and absence of BPA exposure (Fig. 1). To evaluate dose–response, we treated dams with 0, 2.5, 25, 250 or 2500 µg BPA/kg body weight (BW) everyday starting from 2 weeks before conception until the end of the gestational period. Offspring were maintained on control AIN-93G diet for the duration of the experiment. *In utero* exposure to all BPA doses while fed a HBF diet did not affect litter size of the dams or significantly alter the body weights of 2-, 7-, 14-, 21-, 35- and 50-day-old female offspring when compared with either the HBF-alone or control diet groups (data not shown).

Similarly, estrous cyclicity of adult female offspring (data not shown) and serum concentrations of 17β-estradiol, progesterone, leptin and adiponectin in PND21 and PND50 female offspring were not significantly different among control and HBF diet groups (Supplementary Table 3). Interestingly, exposure to HBF plus 2.5 µg/kg BW/day BPA significantly delayed (by ~1.5 days) the onset of puberty in the female offspring when compared with the HBF-alone group, as determined by vaginal opening (Supplementary Table 3). Maternal exposure to a HBF diet alone, however, did not alter the time to vaginal opening in offspring when compared with the control diet group.

**Figure 2:**
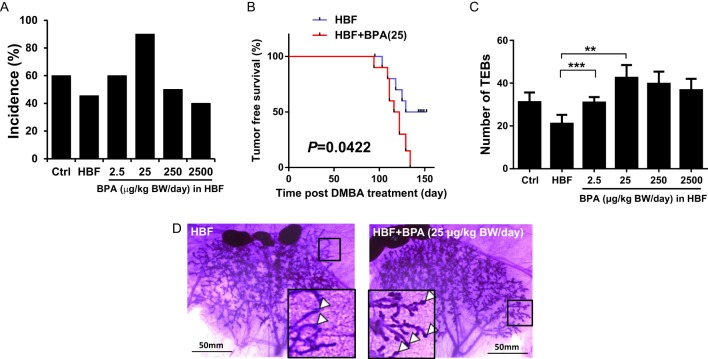
Gestational exposure to high butterfat and bisphenol A increases mammary tumor incidence and effects mammary gland morphology. (A) Tumor incidence (percentage of rats that developed at least one tumor) at PND140, in DMBA-treated offspring fed a control (AIN-93G) diet (Ctrl), or a high-butterfat (HBF) diet in the presence or absence of bisphenol A (BPA) at various concentrations. (B) Time (days) post DMBA treatment to first palpable tumor in HBF-alone vs HBF + BPA25 (µg/kg BW/day) groups (log-rank test, *P* = 0.0422). (C) Number of terminal end buds (TEBs) in PND21 mammary glands, in offspring fed a Ctrl, or a HBF diet in the presence or absence of BPA at various concentrations. Data are expressed as mean ± s.e.m. **P < 0.01, ***P < 0.001 vs HBF, two-way ANOVA. (D) Representative whole mount images of PND21 mammary glands showing TEBs in offspring fed a HBF diet (left) and a HBF diet with 25 µg/kg BW/day BPA (right). Scale bar: 50 mm. Corner inset is a high magnification view of boxed area. Arrowheads mark the location of TEBs.

Using a well-established chemically induced mammary cancer model, we treated female offspring with the carcinogen, DMBA, on PND50 (Russo *et al*. 1979, Heffelfinger *et al.* 2003). Using palpable tumor as the endpoint, we found no significant difference in average time to first tumor appearance (i.e. latency) (Supplementary Fig. 1A), number of palpable tumors (i.e. multiplicity) (Supplementary Fig. 1B) or tumor volume (data not shown) in DMBA-treated rats gestationally exposed to control AIN-93G or HBF diet groups (Supplementary Fig. 1A and B). In contrast, offspring of dams exposed to HBF and 25 µg/kg BW/day BPA during pregnancy had significantly higher mammary tumor incidence (90%) compared with HBF-alone controls (45.5%) (*P* < 0.043) (Fig. 2A) as well as a significantly shorter tumor-free survival time (*P* = 0.0422) (Fig. 2B). Offspring exposed to a control diet with 10% kcal butterfat showed no significant difference in mammary tumor incidence in the absence vs presence of 25 µg/kg BW/day BPA (data not shown).

High-fat intake during pregnancy has been shown to increase offspring’ mammary cancer risk by altering mammary gland development, mainly by increasing the number of terminal end buds (TEBs) (Hilakivi-Clarke *et al*. 1997). We examined the number of mammary gland TEBs on PND21. In contrast to published results using a corn oil high-fat diet (de Assis *et al*. 2012), our HBF group did not show any significant effect on TEB number when compared with the control AIN-93G diet group (Fig. 2C). However, when dams were gestationally exposed to 25 µg or 250 BPA/kg BW/day in addition to a HBF diet, the number of TEBs was significantly increased in the mammary glands of offspring at PND21 (Fig. 2C and D).

### Transcriptome analysis identifies HBF BFBPA-regulated genes associated with cancer-related signaling

**Figure 3:**
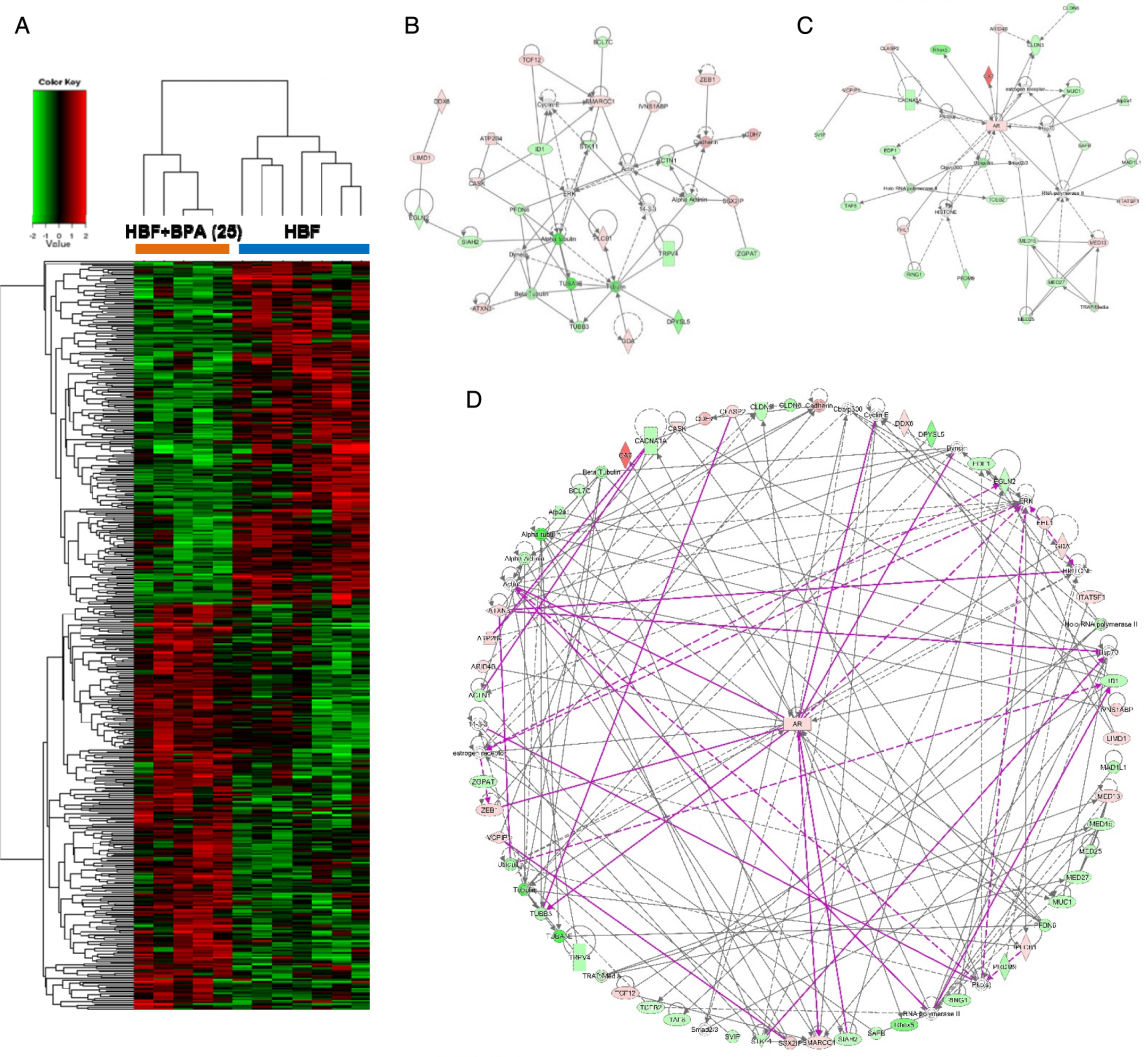
Transcriptome analysis identifies high butterfat intake and bisphenol A exposure-regulated genes associated with cancer-related signaling. (A) Hierarchical clustering analysis of 504 genes with *P* < 0.05 from genome-wide transcription analysis of laser capture microdissected epithelia of PND21 mammary glands from offspring fed a high butterfat (HBF) diet vs a HBF diet with 25 µg/kg BW/day bisphenol A (BPA). Using an unbiased gene clustering method, the heat map shows that differential genes are clearly segregated between samples into two distinct groups. Ingenuity pathway analysis of the 504 genes identified two cancer-related networks associated with the HBF + BPA vs HBF-alone group: (B) extracellular signal-regulated kinase (ERK) rapid signaling and (C) androgen receptor (AR) signaling. (D) Merging of these networks identified AR to be the key node. Green represents low expression; red represents high expression.

To gain insight into how gestational HBF intake in addition to BPA exposure (25 µg/kg BW/day) modulates mammary tumor incidence in offspring, we performed genome-wide transcription analysis on laser capture microdissected (LCM) epithelia of PND21 mammary glands. Using this approach, we identified 504 differentially expressed genes (*P* < 0.05) between the HBF-alone and HBF + BPA (25 µg/kg BW/day) diet groups. Hierarchical clustering clearly segregated the differentially expressed genes into two groups, HBF and HBF + BPA25 (µg/kg BW/day) (Fig. 3A).

To identify biological processes related to the identified dietary exposure-associated genes, we performed pathway analysis by interrogating the knowledge-based Ingenuity Pathway Analysis (IPA) database (Qiagen, www.qiagen.com/ingenuity). Interestingly, the top two networks were found to be cancer related, including ‘Cancer, Cellular development, Embryonic development’ and ‘Gene expression, Cancer, and Organismal injury and Abnormalities’. There were 25 genes involved in each network. One was associated with extracellular signal-regulated kinase (ERK) rapid signaling (Fig. 3B) and the other was related to androgen receptor (AR, Fig. 3C). When these networks were merged, AR appears to be the key node for those two cancer-related networks (Fig. 3D).

Follow-up analysis of the gene expression array study was then performed using qRT-PCR for the ten most upregulated (*Calcb*, *Msl3l2*, *Aldh1b1*, *Spert*, *Astl*, *Magee2*, *Tuba3a*, *LObib52614*, *Cplx4* and *Kcnv2*) and ten most downregulated (*Olr1229*, *Olr791*, *Fam46d*, *Olr788*, *Olr984*, *Olr750*, *Olr51*, *Ccr9*, *Car7* and *Olr830*) genes (Table 1). Notably, there was a panel of seven olfactory receptors (Olr) found to be downregulated by BPA exposure. Of the twenty differentially expressed genes, we found 12 genes (*Aldh1b1*, *Astl*, *Cplx4*, *Kcnv2*, *LOC502684*, *Magee2*, *Tuba3a*, *Car7*, *Olr788*, *Olr830*, *Olr791* and *Olr1229*) were differentially expressed in the amplified LCM samples using qPCR analysis (Supplementary Fig. 2).

**Table 1:** Top differentially expressed genes in mammary glands of offspring gestationally exposed to high-butterfat ± bisphenol A.

			**Putative regulatory CpG island^c^**	
**Gene symbol**	**Gene name**	**Log 2 fold change**	Presence	Location
Upregulated genes				
*Calcb*^a^	Calcitonin-related polypeptide, beta	6.6	No	Not applicable
*Msl3l2*^a^	Male-specific lethal 3-like 2	5.5	Yes	Exon 2
*Aldh1b1*^a^	Aldehyde dehydrogenase 1 family, member B1	5.0	Yes	5′ promoter; TSS; Exon 1
*Spert*^a^	Spermatid associated	4.8	No	Not applicable
*Astl*^a^	Astacin-like metallendopeptidase (M12 family)	4.6	Yes	>5 kb upstream of TSS; 3′ end, >5 kb downstream of Exon 10
*Magee2*^a^	Melanoma antigen, family E, 2	4.3	No	Not applicable
*Tuba3a*^a^	Tubulin, alpha 3A	4.3	No	Not applicable
*LOC502684*	Hypothetical protein LOC502684	3.5	Yes	>10 kb upstream of TSS
*Cplx4*^a^	Complexin 4	3.4	Yes	3′ end; >15 kb downstream of Exon 3
*Kcnv2*^b^	Potassium channel, subfamily V, member 2	3.2	Yes	Exon 1
Downregulated genes				
*Olr1229*	Olfactory receptor 1229	−17.6	No	Not applicable
*Olr791*	Olfactory receptor 791	−9.8	No	Not applicable
*Fam46d*^a^	Family with sequence similarity 46, member D	−8.5	No	Not applicable
*Olr788*	Olfactory receptor 788	−7.3	No	Not applicable
*Olr984*	Olfactory receptor 984	−7.0	No	Not applicable
*Olr750*	Olfactory receptor 750	−6.6	No	Not applicable
*Olr51*	Olfactory receptor 51	−6.6	No	Not applicable
*Ccr9*^a^	Chemokines (C–C motif) receptor 9	−6.4	No	Not applicable
*Car7*^b^	Carbonic anhydrase 7	−6.2	Yes	5′ promoter; TSS; Exon 1
*Olr830*	Olfactory receptor 830	−6.0	No	Not applicable

Genome-wide transcription analysis on laser capture microdissected epithelia of PND21 mammary glands identified 504 differentially expressed genes (*P* < 0.05) between high-butterfat (HBF)-alone and HBF + bisphenol A (BPA, 25 µg/kg body weight/day) diet groups. The top ten differentially up- and downregulated genes are listed.

a Rat genes that are homologous to human genes; ^b^genes with a promoter CG-rich region analyzed using bisulfite sequencing analysis; ^c^the presence and location of the putative regulatory CpG island were predicted by the UCSC Genome Browser.

TSS, transcription start site.

### *In utero* HBF BFBPA exposure dysregulated gene expression in PND21 mammary glands through DNA methylation

We next investigated whether the dysregulation of genes in the mammary glands of HBF + BPA offspring was associated with aberrant epigenetic regulation. The most common epigenetic alteration is methylation of cytosine in CpG dinucleotides in a gene’s promoter region, resulting in alterations of gene expression. Thus, we first checked whether a putative CpG island could be found near the transcription start site of each gene using the rat genome database from The University of California Santa Cruz (RGSC 5.0/rn5) (https://genome.ucsc.edu/cgi-bin/hgGateway). Six of the top upregulated genes (*Msl3l2*, *Aldh1b1*, *Astl*, *LOC502684*, *Cplx4* and *Kcnv2*) showed at least one CpG island within their gene locus (Table 1). In contrast, only one gene of the top ten downregulated genes, *Car7*, has a putative CpG island next to their 5′ regulatory region (Table 1).

**Figure 4:**
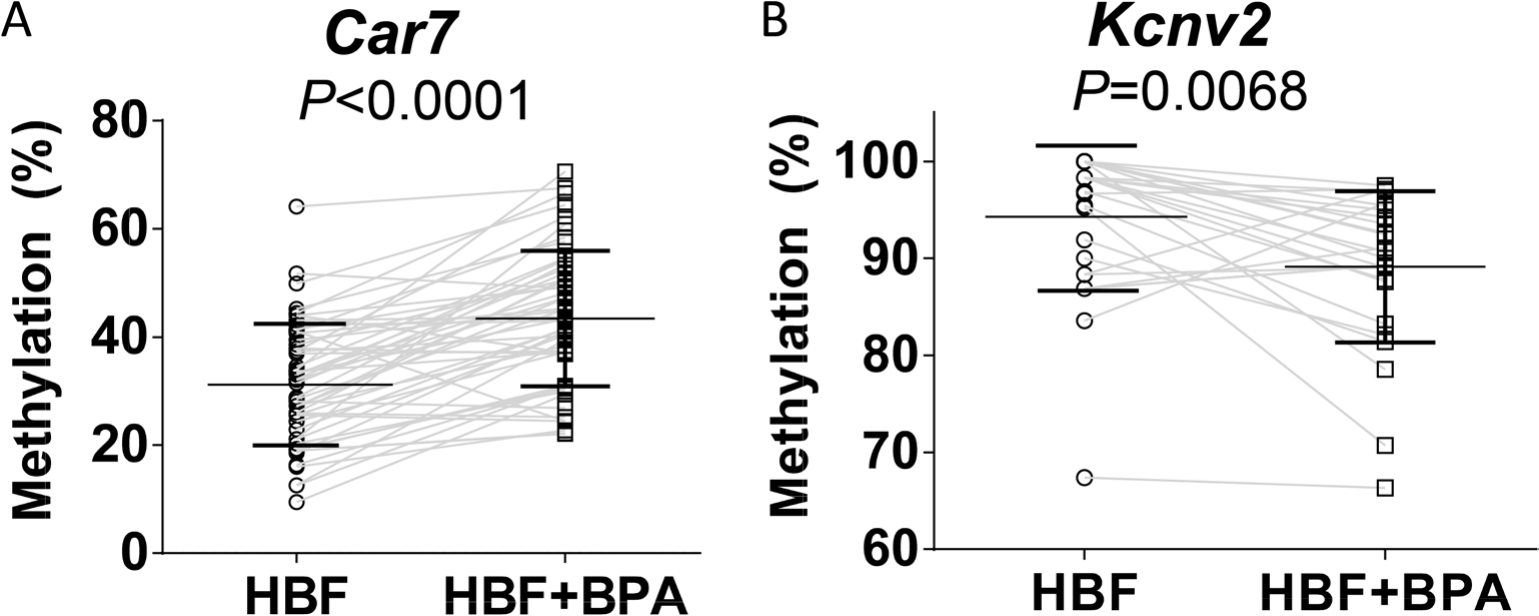
*In utero* high butterfat and BPA exposure alters DNA methylation level of the CpG island in *Kcnv2* and *Car7.* Bisulfite-genomic sequencing was conducted to interrogate differential DNA methylation in (A) *Car7* and (B) *Kcnv2*, in PND21 mammary glands of offspring fed a HBF diet vs a HBF diet with 25 µg/kg BW/day BPA. Percentage of methylation sites in the CpG island of *Car7* (53 CpG sites) and *Kcnv2* (27 CpG sites) were calculated. Each dot represents the average methylation percentage of each site. Mean (middle bar) and standard deviation (upper and lower bars) are represented in each group. Two-way ANOVA was performed to determine the difference between two groups.

We then selected *Car7* (Supplementary Fig. 3), and *Kcnv2* (Supplementary Fig. 4), the only upregulated gene with a CpG island in close proximity to its regulatory region, for bisulfite sequencing analyses. As shown in Fig. 4A, significant hypermethylation was observed in the CpG island of *Car7* in the BPA-exposed group when compared with the control group (*P* < 0.0001, two-way ANOVA). In contrast, the level of methylation in the CpG island of *Kcnv2* was significantly (*P* = 0.0068, two-way ANOVA) reduced after *in utero* exposure to BPA (25 µg/kg BW/day) (Fig. 4B). These methylation changes were inversely correlated with the changes of gene expression as shown in the qPCR experiments (Supplementary Fig. 2).

### Top HBF BFBPA-dysregulated genes are associated with poor overall survival in TCGA breast cancer cohort

**Figure 5:**
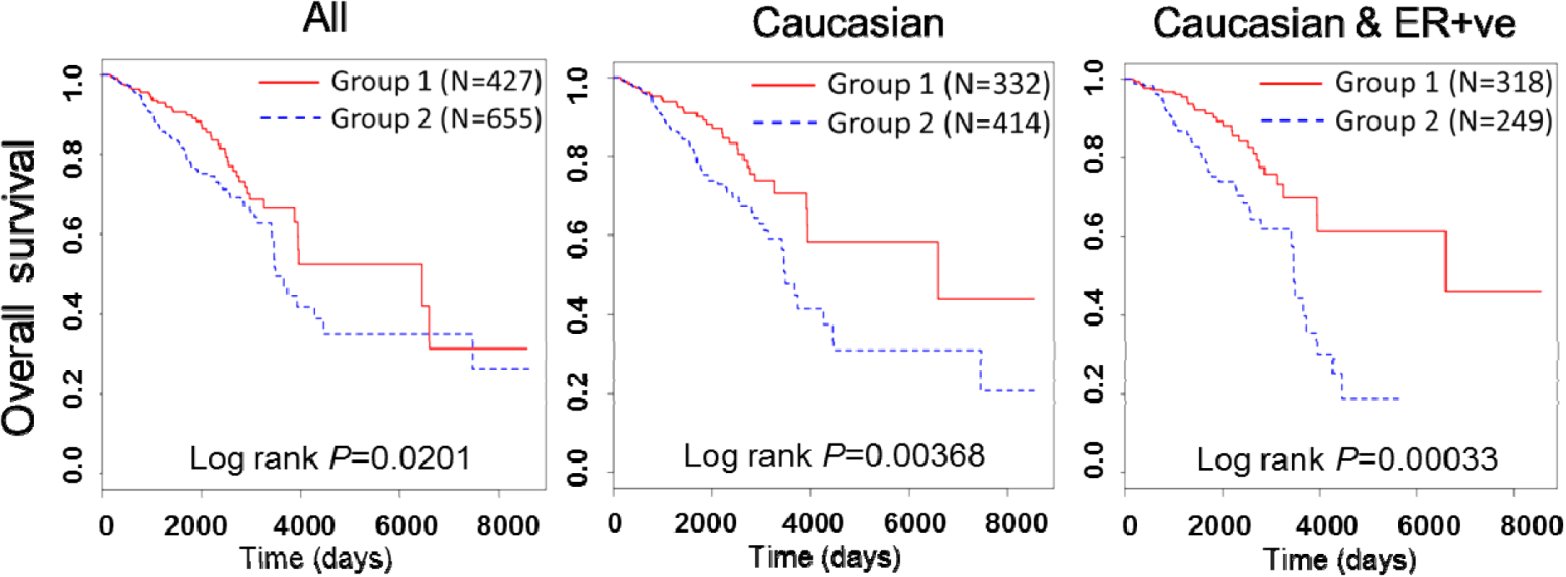
Top high butterfat + bisphenol A-dysregulated genes are associated with poor overall survival in breast cancer patients. Seven differentially expressed BPA genes in TCGA RNA-seq expression data were used to stratify a TCGA breast cancer cohort into two groups using unbiased hierarchical clustering. Survival analyses with log-rank test as well as multivariate survival analyses with Cox’s regression model (adjusted with age at pathological analysis) based on overall survival data available in TCGA were performed. Patients in Group 1 show significantly better overall survival in all patients (left panel), Caucasian patients (middle panel) and Caucasian patients with ER-positive tumors (right panel) compared with group 2.

To gain clinical significance, using The Cancer Genomic Atlas (TCGA) breast cancer cohort, we dichotomized 1082 breast cancer subjects into two groups based on the expression of the seven genes (*ALDH1B1*, *ASTL*, *CA7*, *CPLX4*, *KCNV2*, *MAGEE2* and *TUBA3E*). As it is yet to be determined whether the identified top dysregulated genes are involved in human breast cancer, we selected seven out of the 12 that were clearly defined in humans for survival data analysis in TCGA cohort. Interestingly, we found those seven genes can be used to predict a group of 655 patients (Group 2, Fig. 5 left panel) with poor overall survival in the cohort (*P* = 0.0201). Stratifications on race and patients with estrogen receptor (ER)-positive tumors showed that these seven genes have a better prognostic value in Caucasian patients (*P* = 0.00368, Fig. 5 middle panel) as well as in Caucasian patients with ER-positive breast cancer (*P* = 0.00033, Fig. 5 right panel). Further analysis of Caucasian patients with ER-positive breast cancer suggested that the patients with poor overall survival (Group 2) have significantly less progesterone receptor expression (odds ratio = 3.682, *P* < 0.0001). All other parameters examined including age, lymph node positivity, cancer stage and cancer recurrence showed no statistical difference between the two groups of patients. Interestingly, expression of four genes (*ALDH1B1*, *ASTL*, *CA7* and *TUBA3E*) of the seven gene panel was significantly different between the two groups of human breast cancer subjects (data not shown). The most significantly differentially expressed human genes (*ASTL* and *TUBA3E*) were also found to be upregulated in the poor overall survival group, similar to that of *Astl* and *Tuba3a* in rat mammary glands of the HBF + BPA group.

## Discussion

The primary aim of this study was to determine if maternal exposure to low doses of BPA (2.5–2500 µg/kg BW/day) in combination with a HBF diet modifies mammary gland development and increases the risk of mammary tumorigenesis in first-generation offspring. Our major findings were that as a result of gestational intake of a diet containing 39% kcal from butterfat, exposure to BPA at 25 µg/kg BW/day was most effective in increasing mammary tumor incidence to 90% compared with 45.5% when BPA was not present in the diet. The dose–response curve was non-monotonic and the effective dose was 200-fold and 2000-fold lower than the current no-observed adverse effect level (NOAEL; 5 mg/kg BW/day) and the lowest-observed adverse effect level (LOAEL; 50 mg/kg BW/day), respectively, as established by the US Environmental Protection Agency (EPA) (Agency USEP 2010). Mammary tumor incidence data correlated with significant increases in TEBs observed for the two lowest doses (2.5 and 25 μg/kg BW/day). In fact, in the present study, the BPA dose (25 μg/kg BW/day) that elicited the greatest mammary tumor incidence is 2-fold less than the US EPA’s daily tolerable oral reference dose of 50 μg/kg BW/day for human exposure (Agency USEP 2010), while the lowest effective dose causing increased mammary TEBs is 20-fold lower than the tolerance dose for humans.

In rodent models, BPA has been reported to alter the development of the mammary gland at the biochemical, cellular and tissue levels of organization, in manners suggestive of a heightened risk for mammary carcinogenesis (Markey *et al*. 2001, Munoz-de-Toro *et al*. 2005, Durando *et al*. 2007). Betancourt and coworkers (Betancourt *et al*. 2010) reported that a prenatal BPA dose of either 25 or 250 μg/kg BW/day together with a standard diet had no tumorigenic effect after DMBA exposure at PND50. However, DMBA-induced carcinogenesis at PND100 resulted in a significantly increased number of terminal ducts (Moral *et al*. 2008) as well as a higher incidence of mammary tumors in rats exposed prenatally to 250 μg/kg BW/day BPA (Betancourt *et al*. 2010), suggesting that BPA at 250 μg/kg BW/day could potentially shift the window of susceptibility for chemically induced mammary cancer from PND50 to PND100. To the best of our knowledge, no previous studies have investigated the impact of maternal consumption of a HBF diet together with BPA exposure on breast cancer risk in offspring. In our current study, we observed that concurrent BPA (25 μg/kg BW/day) exposure with a HBF diet background significantly increases mammary tumor incidence in female offspring treated with DMBA at PND50. We also observed significant increases in the number of TEBs in the mammary glands of PND21 rats born to dams fed a HBF diet and 2.5 or 25 μg/kg BW/day BPA during pregnancy. These effective BPA doses that alter mammary gland morphology and cancer susceptibility are 100-fold or 10-fold, respectively, lower than the 250 μg/kg BW/day BPA dose in the previously reported study (Betancourt *et al*. 2010). Additionally, under the HBF diet, the window of susceptibility to DMBA-induced carcinogenesis was reversed back to PND50 in our study. Perhaps, the HBF diet prevents the window-shifting effect caused by BPA, which may make the mammary glands more vulnerable to lower doses of BPA. In addition, independent studies of prenatal exposure to BPA or high-fat diets have observed alterations in the pace at which mammary gland differentiation occurs at different stages of development (Betancourt *et al*. 2010, Hilakivi-Clarke *et al*. 2013, Soto & Sonnenschein 2015). Generally, perinatal administration of EDCs causes accelerated development of the mammary gland associated with increased proliferation and a higher number of TEBs at PND21 (Hovey *et al*. 2005). Therefore, a greater availability of target structures, in addition to a cellular microenvironment favoring carcinogenesis, could explain the increased tumorigenic response (Russo & Russo 1987, Hilakivi-Clarke 2007, de Oliveira Andrade *et al*. 2014).

Contrary to our expectation, we did not observe a higher mammary tumor risk in female offspring born to dams consuming a HBF diet (39% kcal) alone during pregnancy when compared with those born to dams fed an AIN-93G control diet (10% kcal). Furthermore, a trend of reduced mammary tumor incidence (45%) in the offspring exposed *in utero* to a HBF diet vs an AIN-93G control diet (60%) also correlated with a trend of reduced TEB count. Taken together, these findings suggest that offspring born to dams fed a HBF diet were at a reduced risk of developing mammary tumors following DMBA treatment at PND50. Our results are consistent with a previous study (de Oliveira Andrade *et al*. 2014), which observed that exposure to a lard-based high-fat diet during fetal and lactation periods decreases mammary cancer susceptibility in adulthood in rats. Although the source of high fat differed between studies, the fatty acid profile of the high-fat diet used in both studies consisted of saturated (mainly palmitic (16:0) and stearic acids (C18:0)) and monounsaturated (mainly oleic acid (18:1 n-9)) fatty acids, comprising 37% and 38% total fatty acids, respectively (de Oliveira Andrade *et al*. 2014). This is in contrast to published studies that associate *in utero* exposure to a corn oil-based high-fat diet alone, which contains polyunsaturated fatty acids, with an increased susceptibility to mammary tumorigenesis among female offspring (Hilakivi-Clarke *et al*. 1997, de Assis *et al*. 2006, 2012). These results suggest that future studies focusing on dietary fat composition during pregnancy are warranted.

Diet is estimated to contribute to the etiology of 30–50% of all breast cancers; however, the mechanism(s) by which dietary patterns or EDCs modify breast cancer risk is not fully understood (Holmes & Willett 2004, Adebamowo *et al*. 2005, Hilakivi-Clarke 2007). In this study, we showed that gestational exposure to BPA with HBF diet intake dysregulates early cancer-related gene expression even before cancer development, implying that the EDC predisposes cancer risk by reprogramming gene expression in mammary glands. Some of these dysregulated gene events are mediated through epigenetics, DNA methylation in particular. We and others found that alterations in the fetal environment have caused persistent modification in gene expression and susceptibility of disease (Ho *et al*. 2006, Hilakivi-Clarke 2007). Maternal exposure to BPA during pregnancy increases offspring’s risk of prostate cancer and induces hypomethylation of phosphodiesterase type 4 variant 4 (PDE4D4), an enzyme responsible for cyclic AMP breakdown (Ho *et al*. 2006). Furthermore, a high-fat- or ethinyl-estradiol-supplemented maternal diet has been shown to increase mammary cancer risk in several generations of offspring and is associated with changes in the DNA methylation machinery and methylation patterns in mammary tissue (de Assis *et al*. 2012). In the present study, we observed significant change in the methylation status of the promoter region of *Car7* and *Kcnv2*, concomitantly with significant change in their gene expression level, although both genes have never been reported to be breast cancer related in humans. *CA7*, carbonic anhydrase VII, catalyzes the hydration of carbon dioxide into bicarbonate and proton. It is one of the active isoforms found in cytosolic compartment. Although the mechanistic role of *CA7* in cancer is not much known, it has been speculated that the absence of the antioxidant property of *CA7* could contribute to disease progression (Monti *et al*. 2017). Its tumor-suppressive potential was identified in colorectal cancer as reduced *CA7* level was associated with shorter disease-specific survival (Yang *et al*. 2015). *KCNV2*, voltage-gated K+ channel subunit gene family V member 2, can form functional heterotetramers with Kv2 subunits and influence membrane translocation and channel properties (Ottschytsch *et al*. 2002, Czirjak *et al*. 2007). Its role in cancer is largely unclear and only its close relative, Kv9.3, was known to support the growth of colon, lung and uterine cancer cells (Suzuki & Takimoto 2004, Spitzner *et al*. 2007, Lee *et al*. 2015). We also found that a group of olfactory genes in the mammary gland of PND21 female offspring were downregulated in HBF + BPA group when compared with HBF only. Interestingly, the fetal gene expression profile of olfactory receptors has been shown to be modulated by slight nutrient changes in the maternal diet (Rosenfeld 2012).

Altered expression of genes involved in promoting cell proliferation in the offspring’s mammary gland during development of offspring exposed to maternal dietary fat have been found to increase susceptibility to cancer in adulthood (Hilakivi-Clarke 2007). Using Ingenuity Pathway Analysis, we identified the top biological processes related to the identified dietary exposure-associated genes to include ‘Cancer, Cellular development, Embryonic development’ and ‘Gene expression, Cancer, and Organismal injury and Abnormalities’. One was associated with ERK rapid signaling and the other was related to AR. When these networks were merged, AR was the key node for the two cancer-related networks, suggesting that aberrant activation of these signaling pathways may play a key role in adult mammary tumor risk. Interestingly, recent investigations have identified the AR signaling pathway as a target for breast cancer treatment, with several clinical trials currently ongoing (Pietri *et al*. 2016).

In this study, we identified HBF + BPA-related gene dysregulation in developing mammary glands. Although we cannot rule out in the current study that BPA, in the context of a control diet, may dysregulate some of the same HBF + BPA-related genes, offspring exposed to a control diet showed no significant difference in mammary tumor incidence in the absence vs presence of 25 µg/kg BW/day BPA. Pathway analyses suggested that the majority of the identified HBF + BPA-related genes are classified as ‘cancer’ associated, but little information has been reported about their roles in cancer development. We took advantage of the publicly available RNA-seq and survival data from TCGA and determined that seven differentially expressed genes (*ALDH1B1*, *ASTL*, *CA7*, *CPLX4*, *KCNV2*, *MAGEE2* and *TUBA3E*) could be involved in breast cancer development, especially in Caucasian female patients with ER positivity. It is intriguing to see that the ‘signature’, which is composed of 7 genes, could be a significant prognostic indicator for breast cancer patients even though the role of each gene in breast cancer remains largely unknown. Therefore, genome-wide data together with patient-based survival analyses provide an effective way to reveal novel genes/pathways involved in cancer development. However, how those genes are linked to ER signaling and whether those genes functions as oncogenes or tumor suppressor genes in a specific genetic background require more detailed investigation in future.

In conclusion, our data reinforce findings that maternal diet during pregnancy can determine the susceptibility of offspring to the development of breast cancer in adult life (Hilakivi-Clarke 2007, de Assis *et al*. 2012, de Oliveira Andrade *et al*. 2014). Importantly, we found that concurrent exposure to a HBF diet and BPA below the current NOAEL during pregnancy modulates developmental morphology and gene expression in the prepubertal mammary gland and increases the breast cancer incidence in offspring. It is apparent from our findings that the complex interplay of diet and environmental exposure to an endocrine disrupter can reprogram the developing mammary gland permitting a permissive environment for adult breast cancer risk in the first-generation offspring.

## Supplementary Material

Supporting Figure 1

Supporting Figure 2

Supporting Figure 3

Supporting Figure 4

Supporting Table 1

Supporting Table 2

Supporting Table 3

## Declaration of interest

The authors declare that there is no conflict of interest that could be perceived as prejudicing the impartiality of the research reported.

## Funding

This study was supported in part by grants from the National Institutes of Health: U01ES019480, U01ES020988, U54HL127624, RC2ES018765, P30ES006096 and P30ES025128, and the United States Department of Veterans Affairs: 101BX000675.

## Author contribution statement

Experimental design: Y K L, G V, S B and S M H; performing experiments: Y K L, G V, A C, D S, X Z, A K, R G; data analyses: Y K L, G V, A C, D S, X Z, J Y, M M, S M H; writing manuscript: Y K L, G V, A C, J V, J Y, A K, M M, S M H.
