# Supplementary material for: Gestational high-fat diet and bisphenol A exposure heightens mammary cancer risk
Source: Endocr Relat Cancer. 2017 May 9;24(7):345–58. doi: 10.1530/ERC-17-0006 (PMC5488396; doi:10.1530/ERC-17-0006)
Supplement: Supporting Figure 1 [file erc-24-345-s001.pdf]

## Supplementary Figure S1

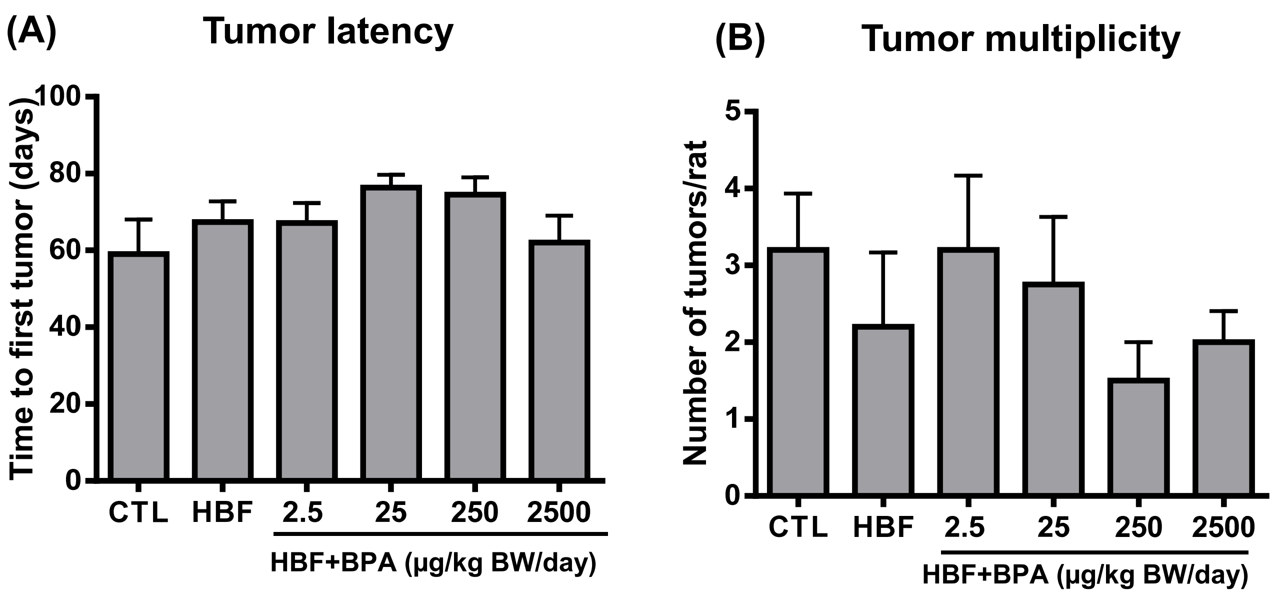


**Supplementary Figure S1**. Tumor latency, and multiplicity. (A) Tumor latency (average time (days) to first tumor) and; (B) Tumor multiplicity (number of palpable tumors per animal), showed no significant difference among control (AIN-93G) diet (Ctrl), or high-butterfat (HBF) diet groups in the presence or absence of bisphenol A (BPA).
