# Supplementary material for: Gestational high-fat diet and bisphenol A exposure heightens mammary cancer risk
Source: Endocr Relat Cancer. 2017 May 9;24(7):345–58. doi: 10.1530/ERC-17-0006 (PMC5488396; doi:10.1530/ERC-17-0006)
Supplement: Supporting Figure 2 [file erc-24-345-s002.pdf]

# Supplementary Figure S2

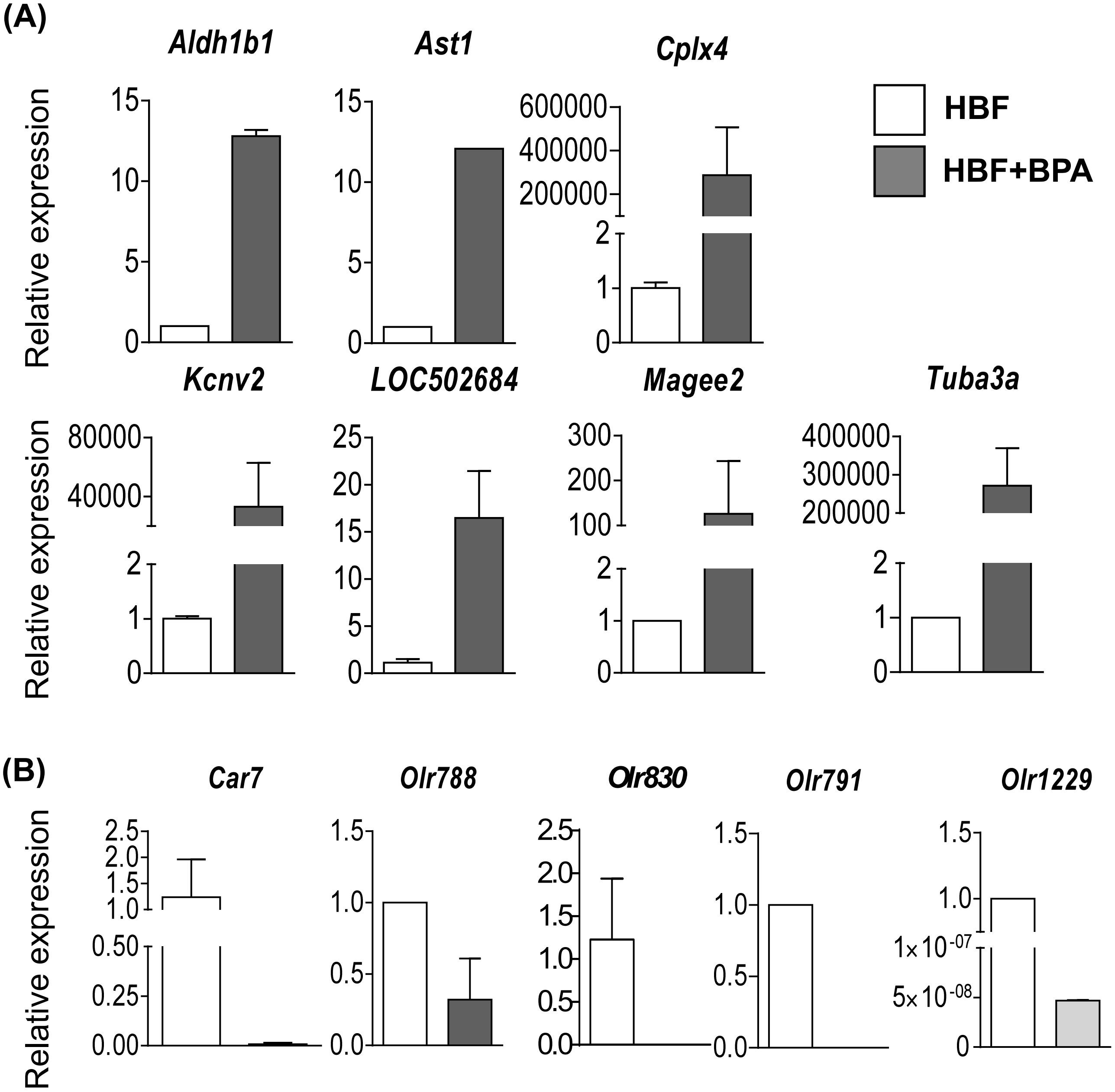


**Supplementary Figure S2. Differential expression of dysregulated genes in mammary glands of offspring gestationally exposed to high-butterfat +/- bisphenol A.** (**A**) Seven up-regulated (*Aldh1b1, Astl, Cplx4, Kcnv2, LOC502684, Magee2, and Tuba3a*) and (**B**) five down-regulated genes (*Car7, Olr788, Olr830, Olr791, and Olr1229*) were detected and measured by real time PCR (qPCR) analysis of microdissected epithelia of PND21 mammary glands from rats gestationally exposed to high-butterfat (HBF) either with or without 25 µg BPA/kg BW/day. A total of n=2-4 samples showed detectable gene expression levels due to low RNA input. Error bar was not shown for samples with very similar relative gene expression levels.
