# Supplementary material for: Gestational high-fat diet and bisphenol A exposure heightens mammary cancer risk
Source: Endocr Relat Cancer. 2017 May 9;24(7):345–58. doi: 10.1530/ERC-17-0006 (PMC5488396; doi:10.1530/ERC-17-0006)
Supplement: Supporting Figure 3 [file erc-24-345-s003.pdf]

Supplementary Figure S3

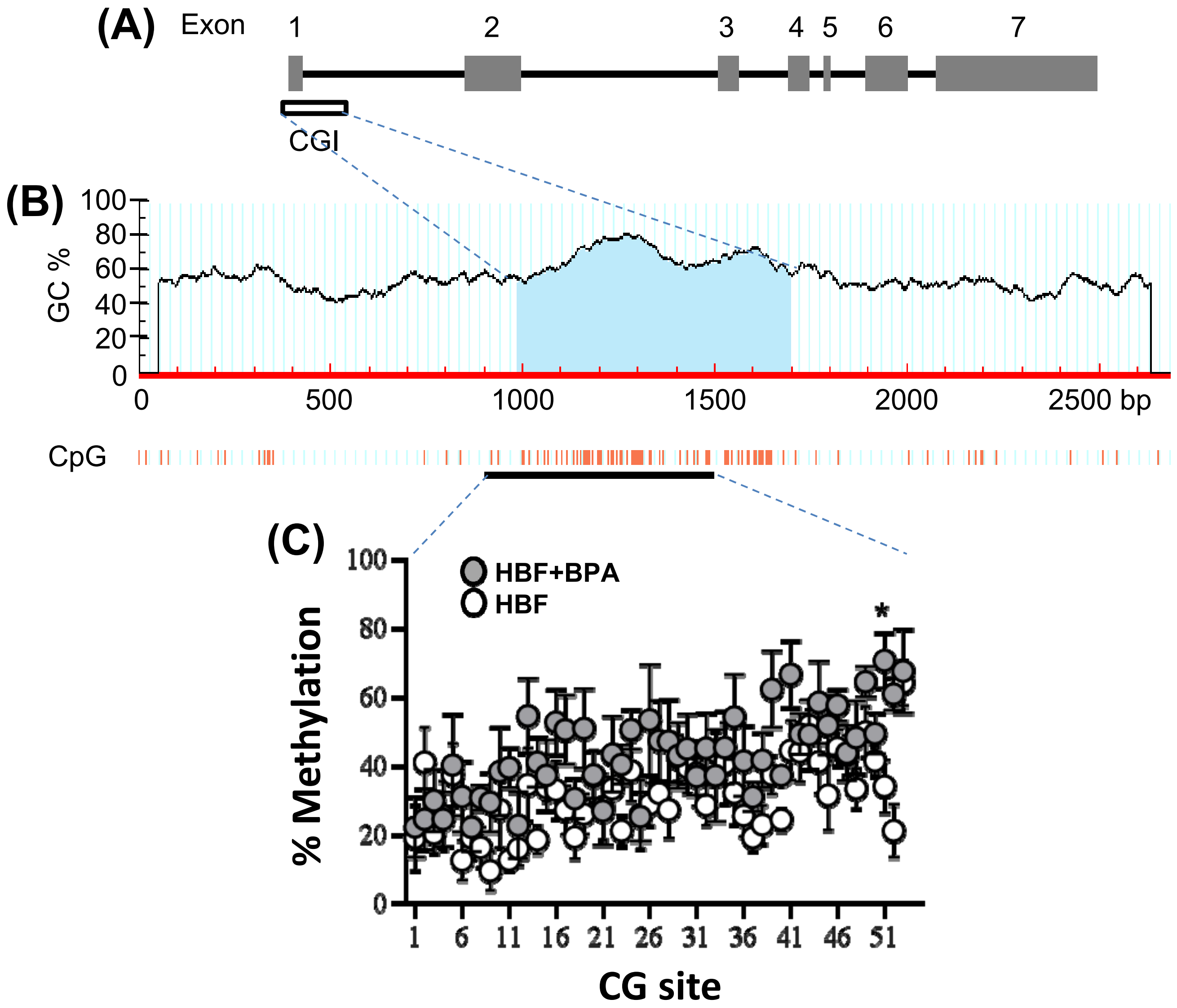

Supplementary Figure S3. Bisulfite sequencing analysis comparing methylation patterns of promoter CpG region of Car7 in mammary gland tissues from rats exposed to HBF and HBF+ BPA. (A) Schematic diagram depicting the location of the CpG island (CGI, white block). Grey blocks indicate the exons and black lines indicate the introns. (B) Predicted CpG region within the promoter of CpG by MethPrimer. The red line indicates the gene sequence at ±1000 bp from the predicted CpG region. The blue region under the curve represents the CpG rich region. The black line indicates the region analyzed using bisulfite sequencing. (C) Promoter methylation pattern analyzed using bisulfite sequencing analysis. Each circle is an average of percentage methylation of a CG site from 4-6 samples, with 8 clones per sample. Data were analyzed by the web-based tool Quantification tool for Methylation Analysis (QUMA) and expressed as percentage (%) methylation per CpG site. Data are expressed as mean ± SEM. ***P<0.001, HBF versus HBF+BPA by Two-way ANOVA.
