# Supplementary material for: Gestational high-fat diet and bisphenol A exposure heightens mammary cancer risk
Source: Endocr Relat Cancer. 2017 May 9;24(7):345–58. doi: 10.1530/ERC-17-0006 (PMC5488396; doi:10.1530/ERC-17-0006)
Supplement: Supporting Table 1 [file erc-24-345-t001.pdf]

Supplementary Table 1. Primers used in qPCR analysis

Gene	Primers (5' → 3')		Amplicon length
Ald1h1	Forward	GCCGCAGGTCCTCAGGATG	79
	Reverse	ATATGAGGTGGTCCTGCCCT	
Ast1	Forward	CTTTGCTCTCCTTGACAGGTTTG	111
	Reverse	ATACTCGGCTTCCCTCAGGA	
Calcb	Forward	AACGCTCCAGGAAGAAGGTTAC	88
	Reverse	CCATGGGGCCTTCTCAATAGG	
Car7	Forward	GACAGAACGGAGTCGGCAG	100
	Reverse	CCAATTTGAAGGGCCGTCGT	
Ccr9	Forward	CTGCACTTCCCCTCCTGAAG	78
	Reverse	TGCCAGGAATAAGGCTTGTGA	
Cplx4	Forward	TGAAGAGAAGATGGAGAGAGATGC	148
	Reverse	CAAATCCACATCGTCCCCAG	
Fam46d	Forward	TCGTACCTCCGCAACCATTT	95
	Reverse	AGGCAGACAGTGCTTTGGTT	
Kcnv2	Forward	TCCTTCTCTGCAGCGGTCTA	100
	Reverse	TAGAGATGCTTACCGCAGCC	
LOC502684	Forward	CCAACCAGTAGCACAGTCCC	125
	Reverse	ACTTCATCAGCCGGACCTTG	
Magee2	Forward	ACGCTACCTAGAATGCAGGC	84
	Reverse	ATTTCAAGGTGAGCCCGAGG	
Msl3l2	Forward	CTGAACCTCTGGCCTTCATATAG	93
	Reverse	GGTCAAGCTGGAAACGCAG	
Olr51	Forward	CTGTCATGTTGCCCGTTGTG	184
	Reverse	AAGATGCAGGGGCAGTCAAA	
Olr750	Forward	GCTAGCAGCCGTATGCTTCA	181
	Reverse	TGTTATGCTGACTGGCCACA	
Olr788	Forward	ACTCATCTCTATGGATTTTGACAGA	118
	Reverse	AAGGCCAACTAACCAGGCAAT	
Olr791	Forward	TGGTGTGGAGATGGTTTTACTTA	92
	Reverse	TTGGGCTCAAAATGGTCAGGT	
Olr830	Forward	GAGGTAAGGATGGAGGCGTG	132
	Reverse	TCCTGTGACTGAGGCCAGAT	
Olr984	Forward	TTGTCCCCATGTTAAACCCCT	73
	Reverse	ACTACACCTTTGAATGCTTGC	
Olr1229	Forward	CAGCCTGATCTCCAGTGTCC	74
	Reverse	GTCAGGTTTCCCACCACAGT	
Spert	Forward	GCACAGGACCTATACCTGGC	121
	Reverse	CGTGCCCCTCTGAGGTAGA	
Tuba3a	Forward	ACTGTGGTCGATGAAGTGCG	118
	Reverse	TGCCAATGGTGTAGTGACCTC	
