# Supplementary material for: Gestational high-fat diet and bisphenol A exposure heightens mammary cancer risk
Source: Endocr Relat Cancer. 2017 May 9;24(7):345–58. doi: 10.1530/ERC-17-0006 (PMC5488396; doi:10.1530/ERC-17-0006)
Supplement: Supporting Table 2 [file erc-24-345-t002.pdf]

Supplementary Table 2. Primers used for bisulfite sequencing.

Gene	Primers (5' → 3')		Amplicon length	Annealing temperature
Car7	Forward	AAGGTGTTTGGTTTATTATGTTTAGGAAAT	650	61
	Reverse	CCTAATTCTCACCCAACTAAATTCACTAC		
Kcnv2	Forward	AGTTGGAATGTTGTGAGTTGGTTAAT	576	58
	Reverse	TAAACCTAAAATCCCCACCACCTATA		
