# Supplementary material for: Gestational high-fat diet and bisphenol A exposure heightens mammary cancer risk
Source: Endocr Relat Cancer. 2017 May 9;24(7):345–58. doi: 10.1530/ERC-17-0006 (PMC5488396; doi:10.1530/ERC-17-0006)
Supplement: Supporting Table 3 [file erc-24-345-t003.pdf]

Supplementary Table 3. Serum hormone concentrations and time to vaginal opening in offspring gestationally exposed to high-butterfat +/- bisphenol A.

	Diet					
	CTL	HBF	HBF+BPA (ug/kg BW/day)			
			2.5	25	250	2500
Serum 17β-estradiol PND21 (ng/mL)	34.58 ± 9.96	28.52 ± 8.13	34.06 ± 11.16	25.81 ± 9.44	42.22 ± 22.49	40.61 ± 8.49
Serum 17β-estradiol PND50 (ng/mL)	46.94 ± 16.00	44.17 ± 20.53	35.88 ± 10.74	36.33 ± 3.48	44.04 ± 9.08	41.87 ± 17.74
Serum progesterone PND21 (ng/mL)	11.57 ± 4.33	12.82 ± 5.73	10.51 ± 5.18	8.12 ± 8.05	14.71 ± 4.89	11.59 ± 4.51
Serum progesterone PND50 (ng/mL)	10.99 ± 5.18	11.92 ± 3.68	13.65 ± 3.90	13.17 ± 3.71	17.55 ± 2.66	12.16 ± 3.64
Serum leptin PND21 (ng/mL)	5.95 ± 0.92	4.41 ± 2.02	5.01 ± 2.95	6.91 ± 3.40	5.30 ± 2.68	3.88 ± 0.51
Serum leptin PND50 (ng/mL)	4.68 ± 1.40	5.95 ± 2.23	3.99 ± 1.05	3.12 ± 1.17	3.69 ± 1.13	2.93 ± 0.76
Serum adiponectin PND21 (ng/mL)	61.41 ± 26.83	69.20 ± 23.99	61.87 ± 19.85	83.70 ± 19.52	89.07 ± 29.33	71.07 ± 41.25
Serum adiponectin PND50 (ng/mL)	3.48 ± 1.43	3.57 ± 2.37	3.80 ± 2.10	3.48 ± 1.79	3.92 ± 0.86	4.25 ± 1.15
Time to vaginal opening (days)	32.83 ± 2.31	32.92 ± 1.61	34.55 ± 2.90*	33.28 ± 2.05	32.50 ± 2.42	33.24 ± 2.13

Ctrl indicates control AIN-93G; HBF, high-butter fat; BPA, bisphenol A; BW, body weight; and PND, postnatal day. **P*<0.05 versus HBF
